# Granulin: An Invasive and Survival-Determining Marker in Colorectal Cancer Patients

**DOI:** 10.3390/ijms22126436

**Published:** 2021-06-16

**Authors:** Fee Klupp, Christoph Kahlert, Clemens Franz, Niels Halama, Nikolai Schleussner, Naita M. Wirsik, Arne Warth, Thomas Schmidt, Alexis B. Ulrich

**Affiliations:** 1 Department of General, Visceral and Transplantation Surgery, University of Heidelberg, Im Neuenheimer Feld 420, 69120 Heidelberg, Germany; clemens.franz@med.uni-heidelberg.de (C.F.); nikolai.schleussner@med.uni-heidelberg.de (N.S.); thomas.schmidt1@uk-koeln.de (T.S.); aulrich@lukasneuss.de (A.B.U.); 2 Department of Visceral, Thoracic and Vascular Surgery, University of Dresden, Fetscherstr. 74, 01307 Dresden, Germany; christoph.kahlert@uniklinikum-dresden.de; 3 National Center for Tumor Diseases, Medical Oncology and Internal Medicine VI, Tissue Imaging and Analysis Center, Bioquant, University of Heidelberg, Im Neuenheimer Feld 267, 69120 Heidelberg, Germany; Niels.Halama@med.uni-heidelberg.de; 4 Department of General, Visceral und Tumor Surgery, University Hospital Cologne, Kerpener Straße 62, 50937 Cologne, Germany; naita.wirsik@uk-koeln.de; 5 Institute of Pathology, University of Heidelberg, Im Neuenheimer Feld 224, 69120 Heidelberg, Germany; Warth@patho-uegp.de; 6 Department of General and Visceral Surgery, Lukas Hospital Neuss, Preußenstr. 84, 41464 Neuss, Germany

**Keywords:** colorectal cancer, adenomas, granulin, GRN, survival

## Abstract

Background: Granulin is a secreted, glycosylated peptide—originated by cleavage from a precursor protein—which is involved in cell growth, tumor invasion and angiogenesis. However, the specific prognostic impact of granulin in human colorectal cancer has only been studied to a limited extent. Thus, we wanted to assess the expression of granulin in colorectal cancer patients to evaluate its potential as a prognostic biomarker. Methods: Expressional differences of granulin in colorectal carcinoma tissue (*n* = 94) and corresponding healthy colon mucosa were assessed using qRT-PCR. Immunohistochemistry was performed in colorectal cancer specimens (*n* = 97), corresponding healthy mucosa (*n* = 47) and colorectal adenomas (*n* = 19). Subsequently, the results were correlated with histopathological and clinical patients’ data. HCT-116 cells were transfected with siRNA for invasion and migration assays. Results: Immunohistochemistry and qRT-PCR revealed tumoral over expression of granulin in colorectal cancer specimens compared to corresponding healthy colon mucosa and adenomas. Tumoral overexpression of granulin was associated with a significantly impaired overall survival. Moreover, downregulation of granulin by siRNA significantly diminished the invasive capacities of HCT-116 cells in vitro. Conclusion: Expression of granulin differs in colorectal cancer tissue, adenomas and healthy colon mucosa. Furthermore, granulin features invasive and migrative capabilities and overexpression of granulin correlates with a dismal prognosis. This reveals its potential as a prognostic biomarker and granulin could be a worthwhile molecular target for individualized anticancer therapy.

## 1. Introduction

Colorectal cancer (CRC) is one of the most common cancer entities, with more than one million new cases per year worldwide, and is the second leading cause of cancer-related death in the western world [1,2,3]. Surgery still offers the only curative treatment option [4]. The five-year relative survival for all patients with colorectal cancer is 65%. Twenty percent of patients with CRC are diagnosed in stage I and 22% in stage II, with five-year relative survival rates of 91% and 82%, respectively. However, survival rates descend dramatically at later stages, so stage IV patients have five-year relative survival rates of merely 12% [5].

Multimodal therapeutic approaches include chemo- and radiotherapy as well as angiogenic inhibitors or immunotherapy and lead to ameliorated survival rates. However, especially for radiochemotherapy, negative side effects like general cytotoxicity, neuropathy, bladder dysfunction and bowel dysfunction, such as chronic diarrhea or radiation proctitis, occur [6,7,8,9,10]. Hence, patient-specific and individualized therapeutic strategies are necessary to further improve survival rates and decrease general toxic side effects of common systemic therapies. Therefore, the investigation of predictive biomarker is crucially focusing on tailored therapeutic approaches. A huge amount of transcription factors, growth factors, oncogenes, knocked down tumorsuppressor genes and cytokines and the tumor microenvironment are involved in the interplay of cancer initiation, progression and metastatic development [11,12,13,14]. Granulin is a growth factor and secreted glycoprotein which arises from cleavage of the precursor progranulin [15,16]. The granulin precursor is located on the long arm of chromosome 17 and contains 13 exons [17]. Cleavage of progranulin occurs through the matrix metalloproteinases (MMPs) 9, 12 and 14, proteinase 3 and neutrophil-secreted elastase [18,19,20,21]. The protease cathepsin L (Cat L) is responsible for degradation of pro-/granulin [22], whereas secretory leucocyte protease inhibitor protein (SLPI) protects it from proteolysis [21]. Under physiological conditions, granulin is expressed in, e.g., neurons, immune cells like macrophages or CD^+^^8^CD^−28^ T cells and epithelial cells [15,21,23,24]. However, granulin exhibits tumorigenic functions promoting cell proliferation, migration, invasion, angiogenesis, immune evasion and cell cycle progression [23,25,26]. Overexpression of granulin or its precursor is found in breast cancer, ovarian cancer, bladder cancer and hepatocellular carcinomas, with an impact on patient survival [26,27,28,29,30]. However, only a few studies have examined the role of progranulin in colorectal cancer [31,32,33].

The objective of this study was to evaluate the potential of granulin as a prognostic factor in colorectal cancer. Therefore, granulin expression was measured in a subset of colorectal cancer specimens, healthy colon mucosa and adenomas. In vitro analysis of HCT-116 cells was performed, in order to assess the migration and invasive properties of granulin. Afterwards, the results were compared with patients’ clinical records and patients’ outcomes.

## 2. Results

### 2.1. Patients’ Characteristics

Ninety-four patients suffering from colorectal adenocarcinoma were included in the study, and received surgery at the University Hospital Heidelberg. Fifteen patients died during follow-up. Mean overall survival of all patients was 63.4 months. Patients aged ≥65 years showed a higher granulin expression. Detailed patients’ characteristics are shown in Table 1.

### 2.2. Expression and Localization of Granulin in Colon Cancer Tissue, Corresponding Healthy Mucosa and Colorectal Adenomas

**Figure 1:**
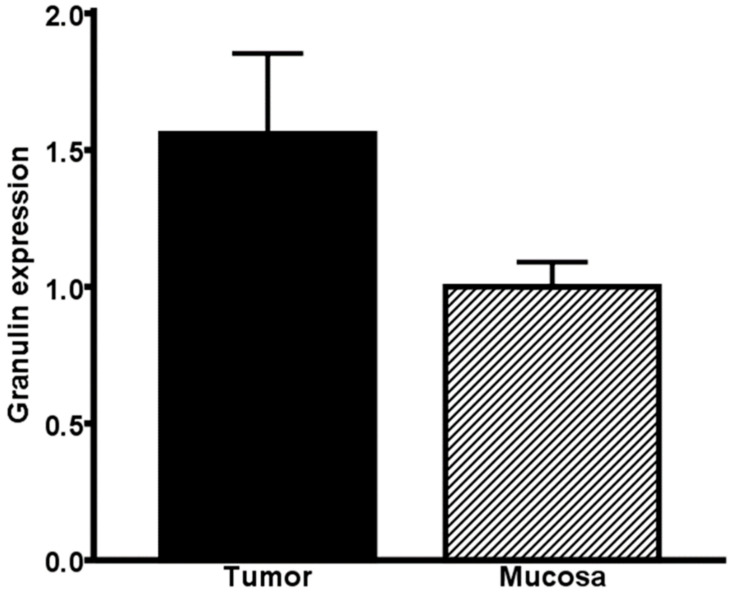
Expression level of granulin. Granulin in tumoral tissue of colorectal cancer specimen and corresponding healthy colon mucosa normalized to 18s rRNA (*n* = 94), (*p* = 0.059). Bar represents mean + SEM.

**Figure 2:**
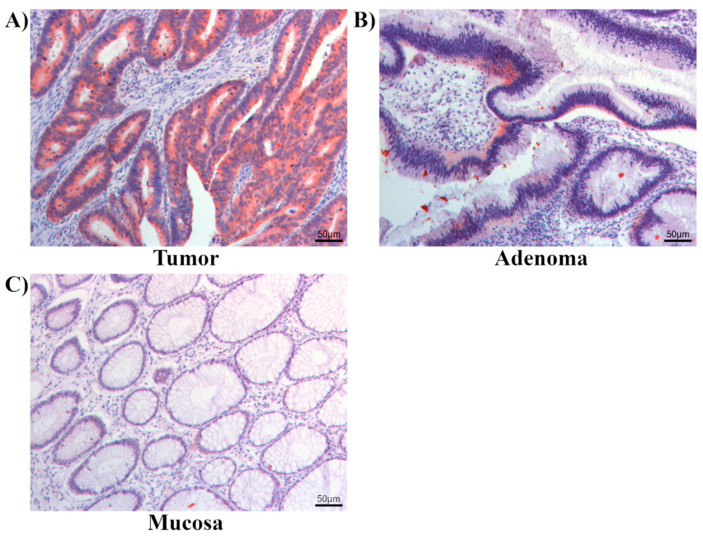
Immunohistochemistry revealing spatial localization of granulin protein. Granulin was strongly expressed in the tumor cells (**A**), particularly in the cytoplasm, and showed a descending expression in colorectal adenomas (**B**), whereas there was almost no expression of granulin in healthy mucosa cells (**C**).

Real-time PCR of colorectal cancer tissues and corresponding healthy mucosa (*n* = 94) exhibited a higher expression of granulin in the tumor tissue than in the healthy colon mucosa (*p* = 0.059) (Figure 1). Immunohistochemical staining was performed in 97 colorectal cancer specimens, 47 corresponding healthy mucosa probes and 19 adenomas. Granulin was mainly and strongly expressed in the tumoral cells—especially in the cytoplasm—of colorectal cancer specimens (94.1%) and showed a descending and weaker expression in adenoma samples (17.9%). Granulin was poorly expressed in healthy colon mucosa (6%) (Figure 2). Consistent with the results of the real-time PCR, the immunohistochemistry revealed that granulin was significantly more highly expressed in colorectal cancer specimens than in healthy colon mucosa (*p* < 0.001). Moreover, granulin was significantly more highly expressed in colorectal cancer specimens than in adenomas (*p* < 0.001). Additionally, granulin was significantly more highly expressed in adenomas than in healthy colon mucosa (*p* = 0.017).

### 2.3. Influence of Granulin on Migration and Invasion of HCT-116 Cells In Vitro

**Figure 3:**
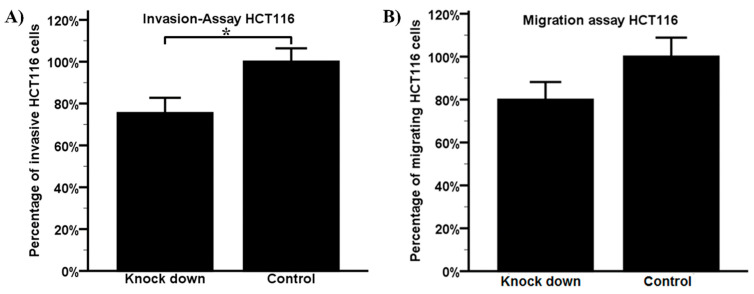
Invasion (**A**) and migration (**B**) assays. Granulin siRNA-transfected HCT-116 cells were less migratory (*p* = 0.074) and significantly less invasive (* *p* = 0.013). Bars represent mean + SEM.

In order to evaluate the impact of granulin expression on the invasive and migration potential, in vitro assays with HCT-116 cells were implemented. After knockdown of granulin with siRNA, we detected a significantly diminished invasive potential of HCT-116 cells (*p* = 0.013), as well as a reduced migration potential (*p* = 0.074) (Figure 3). The mean knockdown efficiency of transfection was 95.6 % (range 95–97%) for the migration assays and 92.3 % (range 85–97%) for the invasion assays in HCT-116 cells measured using RT-PCR. Validation of knockdown efficiency on the protein level using Western blot analysis showed a knockdown efficiency of 84.3%.

In summary, these findings suggest that granulin expression enhances the invasive and migration properties of HCT-116 cells.

### 2.4. Impact of Granulin on Survival

**Figure 4:**
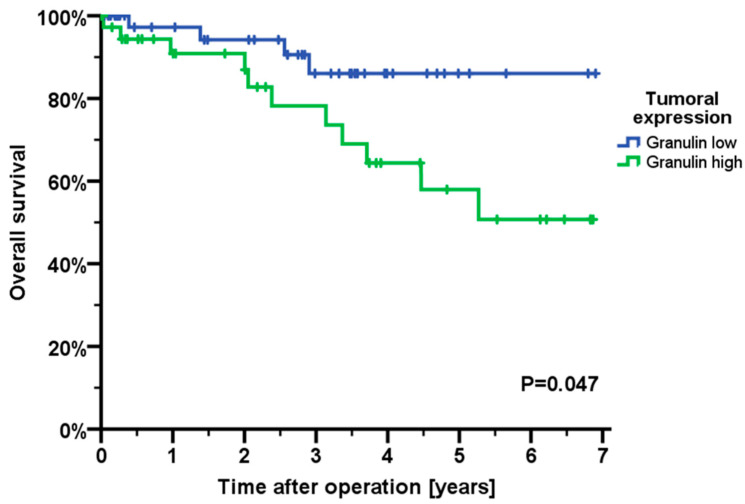
Correlation of granulin expression and overall survival (*n* = 89). Overall survival was significantly impaired in patients with high tumoral granulin expression (*p* = 0.047).

We correlated granulin expression with patients’ overall survival. Therefore, a ratio of the tumoral granulin expression versus the granulin expression of the mucosa was calculated. High expression was defined as tumoral granulin expression higher than in corresponding mucosa, whereas low expression was defined as tumoral expression lower than in mucosa. Patients with a higher tumoral expression of granulin exhibited a significantly shortened overall survival (*p* = 0.047) (Figure 4). Mean overall survival in patients with high tumoral granulin expression was 60.1 months (95 % CI 49.3–70.9 months), whereas mean overall survival in patients with low tumoral granulin expression was 75.6 months (95 % CI 67.9–83.3 months).

Regarding the higher granulin expression in patients aged ≥65 years and the fact that patients with a higher granulin expression exhibit a shortened overall survival, it is tempting to speculate if this effect is due to a potential bias of shortened survival in older patients. In order to exclude this potential bias, we performed further analysis for these subgroups: the median age of patients with high versus low tumoral granulin expression was 66 versus 65 years, and the difference was not significant (*p* = 0.212). Moreover, there was no significant correlation of tumoral granulin expression and age (Pearson’s correlation coefficient ρ = 0.12; *p* = 0.249). Therefore, a potential bias for age and tumoral granulin expression could be excluded.

## 3. Discussion

In the current study, we assessed the impact of the growth factor granulin in colorectal cancer patients. A higher expression of granulin was found in colorectal cancer specimens than in corresponding healthy mucosa with a descending expression pattern regarding colorectal cancer specimens, adenomas and healthy colon mucosa. Moreover, a higher tumoral expression of granulin was associated with worse prognostic outcome. Additionally, in vitro analysis revealed diminished invasive and migration properties of HCT-116 cells. These findings indicate that granulin could be a prognostic biomarker in primary colorectal cancer patients.

Studies have mostly assessed the precursor form of granulin, named progranulin/granulin–epithelin precursor [32,33]. Studies taking the influence of progranulin—especially in colorectal cancer—into account are scarce and, to our knowledge, to date no study has been carried out evaluating granulin itself in colorectal cancer specimens or adenomas. Granulin and the precursor form—which is named differently—acts as a growth factor and mediates cell cycle progression. It is able to complete both S and M phase on its own, remarkably without the need for other growth factors [15]. Moreover, progranulin stimulates proliferation, survival and motility of fibroblasts and epithelial as well as endothelial cells [34,35]. It modulates the immune response, and stimulates migration, invasion, angiogenesis and malignant transformation, revealing its tumorigenic character [23,25,36,37]. An upregulation and tumor-promoting role of granulin or progranulin is commonly found in solid tumors like breast cancer, ovarian cancer or hepatocellular carcinomas [38,39,40]. Pan et al. described an overexpression of granulin–epithelin precursor in tumoral tissues of colorectal cancer patients compared to normal colon tissue which was predominantly localized in the cytoplasm [32]. This is in line with our results revealing an expression of granulin in the cytoplasm of colorectal cancer specimens, as well as a higher expression of granulin in tumoral tissue compared to adenomas and to corresponding healthy colon mucosa. Interestingly, we detected a descending expression of granulin, with the highest occurring in tumoral tissue, less in adenomas and only very weak expression in healthy colon mucosa, indicating a role of granulin in colorectal cancer tumorigenesis.

It is known that HCT-116 cells exhibit high endogenous expression of granulin–epithelin precursor [32]. In our current study, in vitro analysis knockdown of granulin in HCT-116 cells revealed anticancer effects such as inhibition of migration and invasion. Congruently, Pan et al. detected decreased cell proliferation, migration and invasion via the MAPK/ERK signaling pathway in HCT-116 cells after knockdown of granulin–epithelin precursor. Moreover, knockdown resulted in G1 phase arrest of the cell cycle and increased apoptosis [32]. Accordingly, Yang et al. found that progranulin fosters proliferation and angiogenesis through TNFR2/Akt and ERK signaling pathways [33]. Even a role of progranulin as a potential mediator of chemoresistance is described as progranulin levels were higher in the media of chemoresistant colorectal cancer cells [31]. In a nude mouse model of hepatocellular carcinomas, a therapy with a neutralizing antibody against serum levels of progranulin led to inhibition of tumor growth in a dose-dependent manner and reduced angiogenesis in vitro and in vivo [39]. Wong et al. showed in an orthotopic liver tumor model that antiprogranulin treatment inhibits tumor growth, while a combination with cisplatin even eliminated intrahepatic tumors [41]. Additionally, in urothelial cancer cells, a depletion of progranulin reduced tumor cell growth in vivo in xenograft and orthotopic tumor models and suppression of granulin–epithelin precursor sensitized bladder cancer cells for cisplatin [42]. The biological effects of progranulin or granulin indicate the tumorigenic potential, support its role as a biomarker and suggest that its neutralization may be a target for anticancer therapy [23].

In our present study, we detected an association of granulin with overall survival. Patients expressing high tumoral levels of granulin exhibited a significantly impaired overall survival. This is in line with studies in breast, ovarian and hepatocellular cancer. Yeh et al. showed granulin to boost oncogenic STAT3 transcriptional function and correlate with poor prognosis in breast cancer patients [40]. Han et al. assessed progranulin in ovarian cancer patients and described a diminished progression-free survival as well as overall survival in patients overexpressing progranulin [28]. We could not detect an association of granulin and clinical patients’ characteristics, besides age. However, in hepatocellular carcinomas, high progranulin levels were associated with large tumor size and venous infiltration [27].

In summary, we found granulin to be an invasive and prognosis-determining marker in colorectal cancer patients with an ascending expression from healthy colon mucosa to adenomas up to tumoral tissue. However, as a limitation, it has to be mentioned that most of the previous studies assessed the precursor form. Therefore, further studies are needed to evaluate the tumorigenic influence of granulin and its role as a potential biomarker in colorectal cancer patients.

## 4. Material and Methods

### 4.1. Patients and Tissue Samples

Formalin-fixed paraffin-embedded specimens of colorectal carcinomas (*n* = 97), corresponding healthy colon mucosa (*n* = 47) and adenomas (*n* = 19) were recovered from the tissue bank of the National Center for Tumor Disease (NCT), University of Heidelberg, Germany. Fresh frozen tissue of colorectal carcinomas and corresponding healthy colon mucosa from a series of 94 patients was taken immediately after resection and stored at −80 °C until further use. Tissue of healthy colon mucosa was acquired at least 10 cm away from the tumor, so the mucosa could act as a healthy control. All patients underwent surgery at the Department of General, Visceral, and Transplantation Surgery. Written informed consent of all patients was obtained and the study was approved by the local ethics committee (S7082019). Clinical characteristics like gender, date of birth, age, body mass index (BMI), tumor location, histopathological diagnosis including TNM classification and UICC stage, R classification, grading, CEA level, neoadjuvant chemotherapy and overall survival (time from operation up to death or last follow-up) were obtained from each patient.

### 4.2. Isolation of Total RNA and Quantitative Real-Time PCR

Total RNA from tumor tissue and corresponding healthy mucosa was extracted using the RNeasy Mini Kit (Qiagen, Hilden, Germany) according to the manufacturer´s protocol. Reverse transcription was performed using an ImProm-II™ Reverse Transcription System (Promega, Mannheim, Germany). qPCR quantification with LightCycler^®^ 480 SYBR Green I Master (Roche Diagnostics GmbH, Mannheim, Germany) was assessed using five nanograms of cDNA and a Roche Light Cycler^®^ 480 System (Roche Diagnostics GmbH, Mannheim, Germany). Ready to use primers for granulin (Hs_GRN_1_SG QuantiTect Primer Assay, QT00236635) and 18s (Hs_RRN18s_1_SG Quanti tect Primer Assay), both from Qiagen, Hilden, Germany, were acquired. Negative controls were included according to the manufacturer’s instructions using each primer, nuclease-free water and master mix.

### 4.3. Immunohistochemistry

Immunohistochemical staining of 4 µm sections of formalin-fixed paraffin-embedded tissue specimen of colorectal cancer tissue, corresponding healthy colon mucosa and adenomas for granulin (anti-GRN monoclonal antibody, number 2875-1, Epitomics Inc., Burlingame, CA, USA) was performed using a Dako autostainer with a 1:100 dilution. Slides were pretreated at pH 9.0. The average percentage of positive stained cells was scored as follows: 0 (0–10%), 1 (10–25%), 2 (26–50%), 3 (51–75%), 4 (75–100%). The whole tissue of each section was analyzed. Results show the mean percentage of positively stained cells. A board-certified pathologist from the Institute of Pathology, University of Heidelberg performed histopathologic assessment.

### 4.4. Western Blot

Protein lysates were extracted from HCT-116 cells using RIPA buffer with a protease inhibitor cocktail (Roche Diagnostics GmbH, Mannheim, Germany). After measuring protein concentration with a Pierce^®^ BCA Protein Assay Kit (Thermo Fisher Scientific Inc, Rockford, IL, USA), 20 µg protein were added to 6× Laemmli buffer (Bio-Rad, Hercules, CA, USA), denatured and loaded to a 7.5% Mini-Protean TGX Precast Gel (Bio-Rad, Hercules, CA, USA). After electrophoresis at 100V gel was blotted to a nitrocellulose transfer membrane (Bio-Rad, Hercules, CA, USA), blocked and incubated with primary (see immunohistochemistry protocol; dilution 1:1000, and actin antibody, Cell Signaling Technology, Inc., Danvers, MA, USA) and secondary antibodies (goat anti-rabbit, ab6721, Abcam, Cambridge, UK, dilution 1:1000). Chemiluminescence and detection were performed with SuperSignal West Pico Chemiluminescent Substrate (Thermo Scientific, Rockford, IL, USA) and a CCD camera. Data analysis was performed using ImageJ software 1.53 (NIH, Bethesda, MD, USA) and standardized to actin.

### 4.5. Cell Culture and Transfection

HCT-116 colon cancer cells (ATCC^®^ CCL-247™, LGC Standards GmbH, Wesel, Germany) were used for cell culture analysis. RPMI-1640 (Sigma-Aldrich GmbH, Munich, Germany) with 10% fetal bovine serum (Gibco^®^ by Life Technologies, Darmstadt, Germany) 100 U/mL penicillin and 100 µg/mL streptomycin, both from Sigma-Aldrich GmbH, Munich, Germany, was used for preserving HCT-116 cells at 37 °C. According to the manufacturer’s protocol, granulin siRNA (Stealth™ RNAi GRN (HSS178936), Life Technologies GmbH, Darmstadt, Germany) and scrambled siRNA (Stealth™ RNAi Negative Control, Life Technologies GmbH, Darmstadt, Germany) for internal controls were taken and transfected with Lipofectamine 2000 (Life Technologies GmbH, Darmstadt, Germany).

### 4.6. Cell Migration and Invasion Assays

After removal of the Lipofectamine 2000, including medium (Life Technologies GmbH, Darmstadt, Germany), after eight hours, the HCT-116 cells were kept in serum-free medium (RPMI-1640, Sigma-Aldrich GmbH, Munich, Germany) overnight. Thereafter, cell migration (ThinCerts™, 24 well, pore size 8.0 µm, Greiner Bio-One, Frickenhausen, Germany) and invasion assays (BD BioCoat™ Matrigel™ Invasion Chamber, Greiner Bio-One, Frickenhausen, Germany) were applied and performed according to the manufacturer´s protocol. Incubation time of the HCT-116 cells for the cell migration assay was 24 h, and 72 h for the cell invasion assay. Afterwards, the Infinite^®^ F200 Pro Microplate Reader (Tecan, Crailsheim, Germany) at 540 nm was used for spectrophotometry of cell migration and invasion assays. Both assays were carried out three times in triplicate.

### 4.7. Statistics

Statistical analyses were conducted with Excel 2013 (Microsoft Corporation, Redmond, WA, USA) and SPSS version 25 (SPSS, IBM Corporation, Armonk, NY, USA). A paired Student´s *t*-test was used to determine expressional differences of semi-quantitative real-time PCR for granulin in tumors and corresponding mucosa. Expressional data are presented as mean + standard error of the mean (SEM). One-way ANOVA with Tukey’s test as a post hoc test was employed to examine statistical differences in granulin expression in immunohistochemistry for tissues of tumor, adenoma and mucosa. Statistical differences of clinical parameters like gender, age at operation, BMI, M stage, neoadjuvant chemotherapy and CEA were assessed using a Mann–Whitney U test for dichotomous, independent, not normally distributed samples. T stage, N stage, tumor grade and resection margin status were analyzed with a Kruskal–Wallis test for more than two independent samples.

ImageJ software 1.53 was used for Western blot analysis. Median follow up time was calculated as the median difference between time of operation and last follow-up or death of the patient. The Kaplan–Meier method was used to estimate cancer-related overall survival. Differences between survival curves were evaluated by a log-rank test. Results were considered significant at a *p*-value less than 0.05.

## Figures and Tables

**Table 1:** Overview of clinical characteristics and expression of granulin; n/a = not available.

Patient Characteristics	*n*	Median Expression Granulin	IQR	*p*-Value
Gender				0.192
Male	48	0.959	0.559–1.694	
Female	46	0.771	0.445–1.343	
Age at operation				0.03
<median = 65 years	40	0.753	0.435–1.133	
≥ median = 65 years	54	1.012	0.512–1.896	
BMI				0.733
<median = 25.7 kg/m²	34	0.807	0.439–1.432	
≥median = 25.7 kg/m²	34	0.742	0.462–1.433	
n/a	26			
T-Stage				0.453
T1	7	1.035	0.624–1.659	
T2	40	0.76	0.452–1.246	
T3	27	0.758	0.46–1.753	
T4	20	0.959	0.63–1.818	
N-Stage				0.851
N0	44	0.755	0.466–1.43	
N1	22	0.882	0.619–1.26	
N2	28	0.877	0.458–1.78	
M-Stage				0.085
M0	61	0.763	0.454–1.302	
M1	33	0.952	0.618–1.964	
Tumor grade				0.386
G1	1	n/a	n/a	
G2	64	0.815	0.479–1.5	
G3	15	0.841	0.384–1.79	
Resection margin status				0.088
R0	90	0.801	0.462–1.381	
R1	3	2.676	0.953–3.423	
R2	1	n/a		
L-Stage				0.571
L0	76	0.859	0.479–1.597	
L1	18	0.741	0.455–1.34	
V-Stage				0.24
V0	85	0.801	0.462–1.405	
V1	9	1.189	0.605–2.247	
Neoadjuvant therapy				0.318
Yes	80	0.835	0.479–1.65	
No	14	0.785	0.386–1.22	
Preoperative CEA				0.394
≥2.5 µg/L	54	0.895	0.497–1.469	
<2.5 µg/L	35	0.747	0.457–1.659	

## Data Availability

The data presented in this study are available on request from the corresponding author.The data are not publicly available due to privacy restricitions.
